# Effect of Anionic Polyacrylamide Polymer on Frost Heave Mitigation and Its Implication for Frost-Susceptible Soil

**DOI:** 10.3390/polym15092096

**Published:** 2023-04-28

**Authors:** Yukun Ji, Haihang Wang, Xiaozhao Li, Peng Zhao, Qinke Wang, Ruilin Li, Veerle Vandeginste

**Affiliations:** 1 State Key Laboratory for Geomechanics and Deep Underground Engineering, China University of Mining and Technology, Xuzhou 221116, China; 2 Yunlong Lake Laboratory of Deep Underground Science and Engineering, Xuzhou 221116, China; 3 School of Civil Engineering and Architecture, Southwest University of Science and Technology, Mianyang 621010, China; 4 Department of Materials Engineering, KU Leuven, Campus Bruges, B-8200 Bruges, Belgium

**Keywords:** anionic polyacrylamide polymer, frost heave, ice lens growth, seasonally frozen soil

## Abstract

Seasonally frozen ground regions occupy approximately 55% of the exposed land surface in the Northern Hemisphere, and frost heave is the common global problem in seasonally frozen soil areas. Frost heave induces uneven deformation of ground and damages railways, road paving, and buildings. How to mitigate frost heave is the most important technical issue in this field that has provoked great interest. Here, using freezing experiments, we investigate the effect of anionic polyacrylamide (APAM) polymer on frost susceptible soil. The results demonstrate a so-far undocumented inhibition of frost heave by APAM in freezing soil, namely APAM (tested at concentrations from 0.0 wt% to 0.60 wt%) slows down the frost heave by a factor of up to 2.1 (since 0.60 wt% APAM can decrease frost heave from 8.56 mm to 4.14 mm in comparison to the control experiment). Moreover, it can be observed that the maximum water content near the frozen fringe decreased from 53.4% to 31.4% as the APAM content increased from 0.0 wt% to 0.60 wt%, implying a mitigated ice lens growth. Hydrogen bonding between APAM and soil particles triggers an adsorption mechanism that accumulates soil particles, and thus can potentially inhibit the separation and growth of the ice lens. Moreover, the residue of APAM due to hydrogen bonding-induced adsorption in the pores of granular media may narrow seepage channels (capillary barriers) and provide an unfavourable condition for water migration. The use of APAM can also increase the viscosity of the solution, which causes a greater water migration resistance. This research provides new insights into APAM-influenced frost heave (introducing APAM into the soil can induce bridging adsorption between APAM polymer segments and a particle surface), can enable engineers and researchers to utilise chemical improvement design and to consider suitable actions (e.g., by injecting APAM solution into a frost susceptible soil or using APAM-modified soil to replace the frost susceptible soil) to prevent frost heave from having a negative impact on traffic roads and buildings in cold regions.

## 1. Introduction

Frost heave is the main factor that can cause severe damage to railways, roads and buildings in seasonally frozen soil regions, decreasing the long-term performance of those infrastructures [1,2]. Considerable efforts have been made to mitigate frost-heave-induced geo-hazards [3,4]. With the propagation of the freezing front (approximately 0 °C) in cold climates, in situ water freezes and external water migrates upwards to feed the growth of the ice lens (frost heave) [5,6,7]. Long-term frost heave and the uplift on the ground surface augment geotechnical risk. During warm periods, soil softening occurs during thawing because of the phase transition from ice to liquid, and thus it can result in non-uniform settlement. In Northern China, approximately 80% of irrigation canals (2.4 million km) located in seasonally frozen soil regions are suffering varying degrees of destruction, and the annual maintenance costs in some provinces can reach up to USD 1 million [8]. It has been reported that the potholes and frost heaves induced damages to bridges and roads cost hundreds of millions of dollars annually in the USA [9]. Hence, the soil improvement techniques for water migration control and frost heave mitigation have led to increasing concern [10,11,12].

It is noted that previous research studies provide us with a fundamental understanding of frost heave mitigation. It has been demonstrated that the addition of Portland cement and fly ash can reduce the frost susceptibility of soil [13]. It has been reported that cement in a range from 9% to 13% is proven effective in mitigating frost heave of loess soil (widely distributed in cold regions) [14], and lower frost heave can be expected if the added fly ash content is less than 15% [15]. The cement and fly ash are effective in mitigating frost heave due to the hydration of the active compounds (e.g., calcium silicates and calcium aluminates), these cementitious materials can bond soil particles (enhance soil strength) and decrease permeability. However, various toxic metals and ions leaching from fly ash–soil mixtures are documented in wet environments, and serious environmental problems and risks can emerge [16,17]. In addition, it is noted that the cement is mainly composed of calcium aluminium silicate, whereas a trace amount of Cd, Pb, Ti, Fe, and silica can be found leaching from cement [18]. Long-term heavy metal mobilisation from cement may result in environmental risks. Moreover, the deposition of cement dust on the soil surface and its penetration into deeper soil layers can also impact soil fertility, and the stress can persist in agricultural soils [19]. Phase change materials (e.g., paraffin) may have a positive impact on reducing temperature variation due to the release of latent heat (during the phase transition from liquid to solid); however, loss in compressive strength is reported [3]. Hence, a novel alternative method is required to propose and control frost heave-induced geohazard risks.

The APAM has the characteristics of low carbon and no toxicity. The role of polyacrylamide in each of steep slope treatment, highway cuts stabilisation, and other disturbed soils is striking since it can stabilise soil aggregates and is environmentally friendly [20,21,22]. Moreover, microstructural studies demonstrate that the attachment of APAM onto clay particles promotes the formation of a stable soil structure [23]. It has been documented that the adsorption of polyacrylamide polymer onto the surface of soil particles occurs via hydrogen bonding between the silanol and aluminol OH groups at the particle surface and the polyacrylamide’s primary amide functional groups [24]. A few attachment sites enable the adsorption of polyacrylamide polymer onto soil particles, and the bulk of the polyacrylamide chains can adhere to adjacent soil particles. Hence, the high affinity of polyacrylamide to soil particles can be ascribed to the multi-segment adsorption of its long chain. This explains the aggregation of soil particles and the enhanced cohesion using high molecular polyacrylamide polymer. Moreover, silty clay (often defined as frost susceptible soil) tends to adsorb more polyacrylamide polymers (higher aggregation of soil particles is expected) than coarse-textured soil since it has a higher accessible surface area [25]. However, no previous research has been reported on the impact of APAM on the mitigation of frost heave in frost susceptible soil, and whether APAM can mitigate frost heave and the mechanisms behind the inhibition of water migration (ice lens growth) remain poorly understood. Given polyacrylamide polymer adsorbed onto soil particles may narrow the pore diameter (decreased seepage channel) and enhance soil strength, in this paper freezing experiments were designed and carried out to investigate frost heave considering soil treatment with different APAM contents. The results obtained will enable new insights into ice lens growth in frozen soil in the presence of APAM (i.e., increased fluid viscosity, narrowed seepage channel, and enhanced soil strength), thus providing engineers with a chemical engineering method (e.g., by injecting APAM solution into a frost susceptible soil (low-energy injection method is suggested for introducing APAM into the soil for potential engineering applications [26]) or using APAM-modified soil to replace the frost susceptible soil) for the treatment of frost susceptible soil.

## 2. Methodology

### 2.1. Experimental Material

The soil used in this study is silty clay sourced from Tibetan Plateau, this soil is recognised as frost susceptible soil, and the mineral composition is shown in Table 1. The soil samples were vacuum saturated, and an ultimate water content of 25% was achieved. The detailed physical properties of this silty clay are shown as follows (Table 2). It is noted the APAM powder was added to deionised water in a beaker, and the beaker was placed on a hot plate stirrer. A constant temperature of 40 °C was used to dissolve APAM since it was found this polymer did not fully dissolve at 25 °C. Upon the complete dissolution and cooling down to room temperature, different contents of APAM solution were used to mix with the silty clay. Furthermore, 0.05%, 0.15%, 0.30%, 0.45%, and 0.60 wt% APAM (weight percent, the ratio of PAM weight to soil weight) were evenly mixed with soil to form chemically modified soil samples. The dimensions of these soil samples were 100 mm × 100 mm × 210 mm, and a petroleum jelly pre-treated Plexiglas cube was used as the experimental vessel for the well-prepared soil samples.

### 2.2. Experimental Apparatus

**Figure 1:**
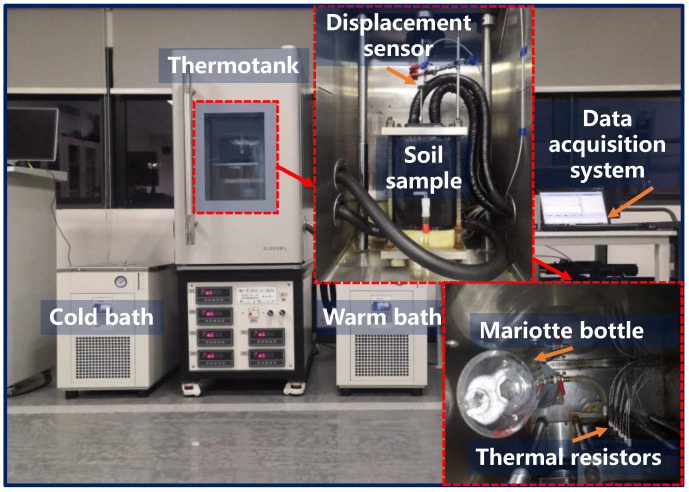
Frost heave testing apparatus.

A frost heave experimental system is applied to examine APAM performance in frost heave mitigation. It consists mainly of a freezing measurement system, temperature system, water supply system, and data collection system (Figure 1). The freezing measurement system is composed of a displacement sensor and thermal resistors (temperature sensors). The temperature system consists of a cold bath, a warm bath, and a thermotank. The water supply is mainly composed of a Mariotte bottle (water supply tube), which can provide external water to feed the growing ice lens during freezing. The intelligent data collector and the corresponding data collection software constitute the data collection system. Moreover, an NDJ-8S rotary viscometer was employed to measure the viscosity of APAM solution at room temperature, and the permeability of the APAM-modified soil was determined by a TST-55 permeameter at room temperature.

### 2.3. Experimental Procedures

(1) Assembly of the sample. Firstly, the soil samples were installed into the Plexiglas cube, and the cold end which can freeze the soil sample from top down was placed at the top of the soil sample. Secondly, the displacement sensor (measurement range of 50 mm) was applied at the top of soil sample. Moreover, eight thermal resistors embedded along the soil sample (at different heights of 15 mm, 40 mm, 65 mm, 90 mm, 115 mm, 140 mm, 165 mm, and 190 mm) were applied to monitor temperature.
(2) A thermostatic process. Both the warm end and cold end were maintained at 5 °C (500 min) until the temperature was longitudinally and uniformly distributed along the soil samples. It is noted that the thermotank was switched on and controlled at a constant temperature of 5 °C in the meantime.
(3) Soil freezing process. The warm end of the soil sample and thermotank were continuously kept at 5 °C, and the cold end initiated a cold temperature of −20 °C. The cold end temperature can subsequently enable a progressively downward movement of the freezing front.
(4) Data logging. Data taker loggers were used to collect the temperature at different positions of the soil samples and to collect the movement of soil surface (frost heave). These measurements occurred at 10 min intervals during freezing. After frost heave experiments, the soil columns were sliced into several soil columns of 1 cm in height, and water distribution was subsequently determined using a drying method (using a thermostat drying box).

## 3. Results

The impact of APAM on frost heave is investigated using one-dimensional freezing tests in this study, and the viscosity, permeability, shear strength, scanning electron microscope (SEM), and Fourier transform infrared spectroscopy (FTIR) analyses are subsequently conducted to investigate the underlying physicochemical mechanisms related to frost heave mitigation, fundamental to our understanding of a wide range of processes of water migration, ice lens growth, and frost heave control.

### 3.1. Frost Heave Test

#### 3.1.1. Temperature and Temperature Gradient

**Figure 2:**
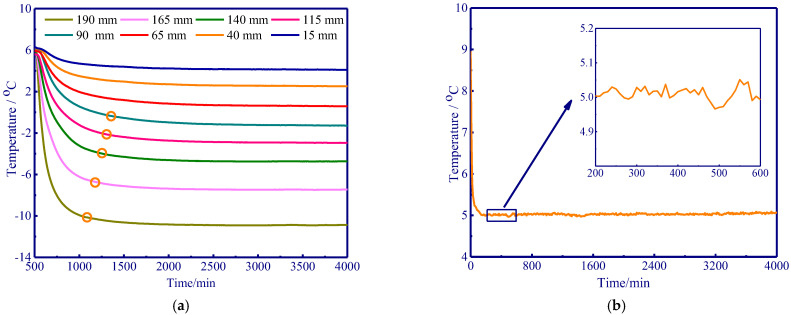
Schematic of temperature distribution and ambient temperature. (**a**) Temperature field; (**b**) ambient temperature.

The temperature gradient enables a pressure gradient, which adsorbs external water from the warmer portion to feed the water loss at the ice particles. The temperature gradient is the main driving force for water migration, and it is closely related to the growth of the ice lens. The temperature distribution along the soil column may play an important role in determining the temperature gradient at the different positions in the soil. At the initiation of freezing, the temperature at different positions in the soil maintained a temperature of 5 °C (Figure 2a). Moreover, the ambient temperature slightly varied during freezing and the temperature in the experimental set-up can be well controlled at 5 °C (Figure 2b). Cold temperature penetrates into the soil from the top down, and a stable temperature field inside the soil column was ultimately formed after approximately 1500 min. Additionally, orange circles were used to record the initiation of a stable temperature at different positions in the soil, and the soil temperature dropped and stabilised faster for positions in closer proximity to the cold end (Figure 2a). 

Given the temperature gradient can dominate the frost heave rate [27], the variation of temperature gradient and frost heave rate was demonstrated, as well as their relation. It is noted that the temperature gradient denotes the relative variation of temperature (ranging from the cold end of the soil sample to the advancing freezing front), and it can be determined as follows:

(1) $$grad\;T=(T_{c}-T_{f})/D$$

where *T_c_* denote the temperature of the cold end, *T_f_* represents the freezing front temperature, and *D* is the distance from the cold end and the freezing front.

Furthermore, the frost heave rate can be determined by the following equation:

(2) $$V_{t}=\frac{h_{2}-h_{1}}{t_{2}-t_{1}}=\frac{\Delta h}{\Delta t}$$

**Figure 3:**
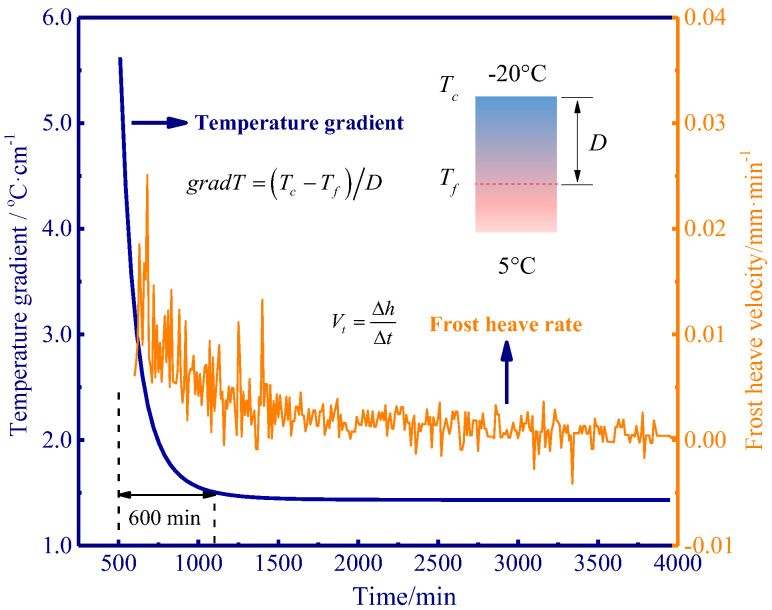
Schematic of temperature gradient and frost heave rate.

Given the pattern of frost heave rate in different experiments showed a similar trend to one another, the control experiment (0.0% APAM) was selected to analyse the variation of frost heave rate during freezing. At the initiation of freezing, a high-temperature gradient was observed, and it rapidly dropped and reached a stable state after 600 min. A higher temperature gradient provided a greater pressure gradient which resulted in significant water flow and ice lens growth [28]. The frost heave rate was the response to the temperature gradient, a higher temperature gradient induced a higher frost heave rate, and a slight change in frost heave rate corresponded to a smaller temperature gradient (Figure 3).

#### 3.1.2. Frost Heave Using Different Contents of APAM

**Figure 4:**
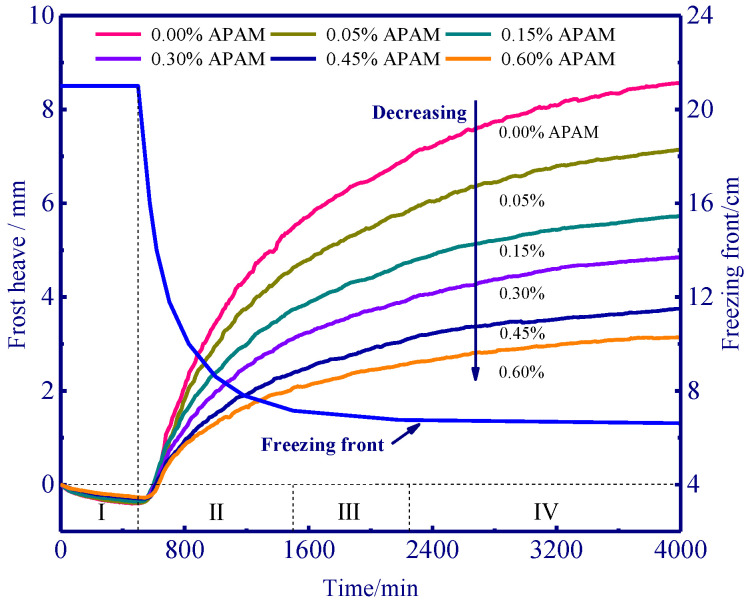
Frost heave with respect to different contents of APAM.

Similarly, the pattern of frost heave development demonstrated a similar trend to one another, and it can be roughly distinguished into four stages (Figure 4) [29]. In a thermal equilibrium stage (I), we observed a shrinkage deformation since the advancing freezing front can increase pore pressure and expel the liquid water, thus leading to the deformation of the soil skeleton. After 500 min, a rapid development stage of frost heave (II) can be observed as the cold energy penetrated and the freezing front advanced, and an obvious increase in frost heave can be observed. After 1500 min, the heaving velocity slightly decreased as frost heave entered a transitional development stage (III). Moreover, a stable stage of frost heave can be expected after 2500 min, the heaving velocity dropped and only a slight increase in frost heave can be observed in this stage. In the control experiment (0.0% APAM), an ultimate frost heave of 8.56 mm was achieved after 4000 min, and this frost susceptible soil can be classified as a strong frost heave level according to the code for design of soil and foundation of a building in frozen soil region of China JGJ 118-2011 (JGJ 118-2011, [30]). Most importantly, frost heave can be inhibited to 4.85 mm (43.3% decrease) if 0.30% APAM was used in the soil. Hence, 0.30% APAM can slow down the frost heave by a factor of 1.8 (8.56 mm divided by 4.85 mm), and the chemically modified soil using APAM can then be determined as a weak frost heave level. Furthermore, 0.60% APAM was found more effective in mitigating frost heave, and it slowed down the frost heave by a factor of 2.1 (8.56 mm divided by 4.14 mm). These results validate the effectiveness of APAM for frost heave control in cold regions.

**Figure 5:**
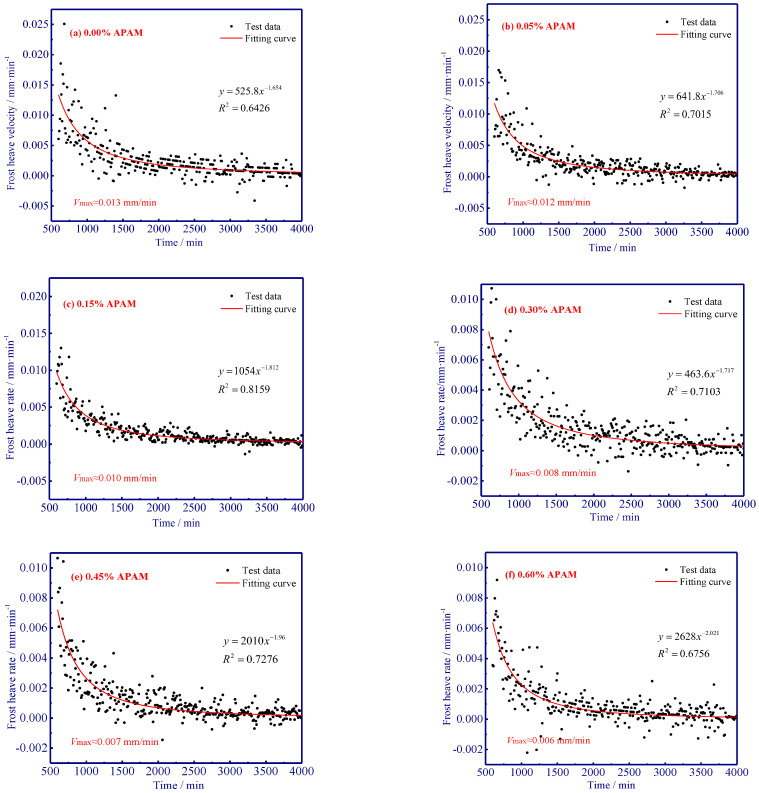
Frost heave velocity with respect to different contents of APAM.

In addition, discrete points that can represent the observed frost heave velocity in different APAM experiments were applied to quantitatively evaluate APAM effects on frost heave, and power functions were used for curve fitting of APAM-influenced frost heave velocity (Figure 5). Our results show that the highest frost heave velocity was observed at the initiation of freezing because of the high-temperature gradient (similar results can also be found in Zhou et al. [31]), and it slowed down as the freezing propagated. Moreover, the observed frost heave velocity (at 600 min) was determined as the maximum frost heave velocity and selected to compare different frost heave velocities in different APAM content experiments. It was found that the maximum frost heave velocity (*t* = 600 min) decreased from 0.013 mm/min to 0.006 mm/min as the use of APAM increased from 0.0% to 0.60% (Figure 5).

#### 3.1.3. Distribution of Water Content

**Figure 6:**
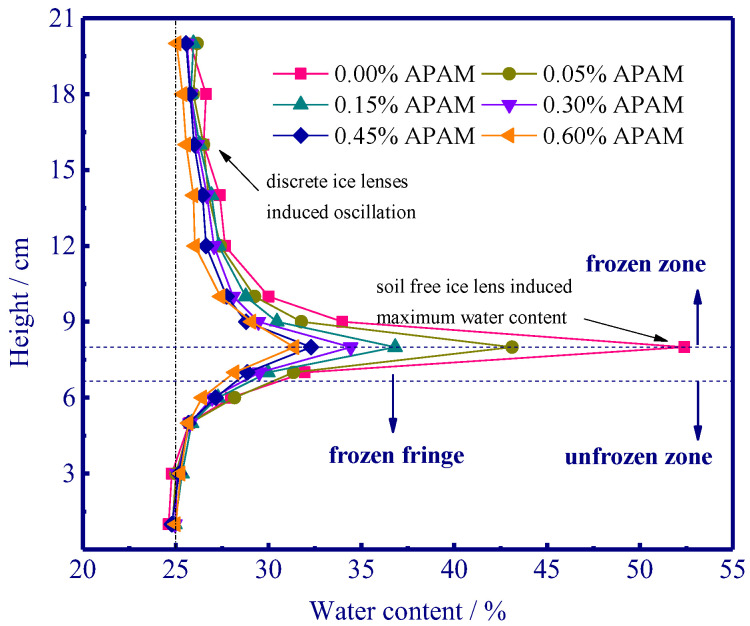
Water distribution along the soil column.

The drying method was used to evaluate the water distribution along the soil column. The initial water content is approximately 25%, and significant water distribution occurred after freezing (Figure 6). The maximum water content related to the formation and growth of the thickest (warmest) ice lens was observed near the freezing front [32]. Furthermore, the discrete ice lenses were discontinuously distributed, and the oscillation of water was ultimately observed in the frozen zone since the newly emerged ice lens can block the water flow to the previously growing ice lens until the warmest ice lens formed (external water can migrate into the frozen fringe and feed the growth of the warmest ice lens). Additionally, the maximum water content decreased as APAM content increased, and less water redistribution was observed with an increase in APAM content. It can be observed the maximum water content near the frozen fringe decreased from 53.4% to 31.4% as APAM content increased from 0.0% to 0.60%.

#### 3.1.4. Shear Strength of APAM-Modified Soil

**Figure 7:**
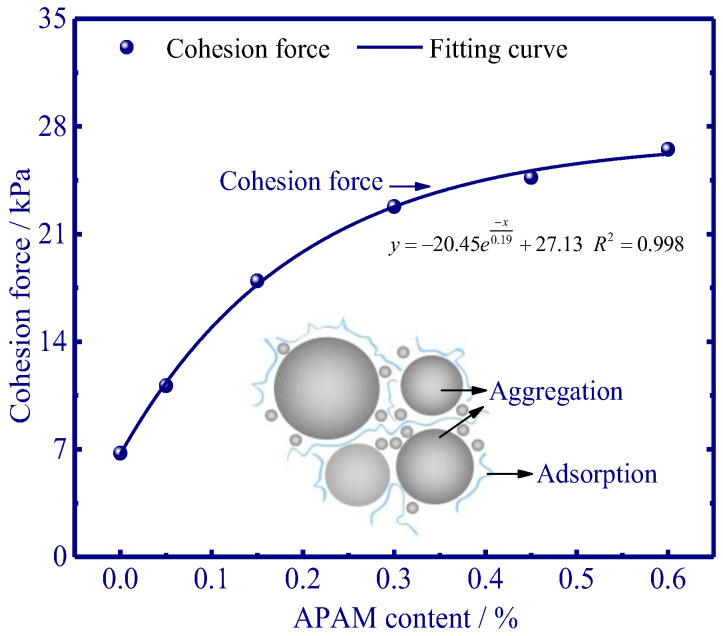
Cohesion force with respect to different contents of APAM.

An exponential function was used for curve fitting of shear strength experiments, and the R-squared value for the cohesion force curve was 0.998 (Figure 7). Higher APAM content is closely related to the higher cohesion force of silty clay, and a content of 0.60% APAM can contribute a 20 kPa increase in cohesion force. Moreover, the development pattern of cohesion force tended to stabilise if the APAM content was higher than 0.30%. A higher cohesion force also implies an enhanced aggregation [33], and thus it can potentially hinder ice lens emergence.

#### 3.1.5. Viscosity and Permeability of APAM-Modified Soil

**Figure 8:**
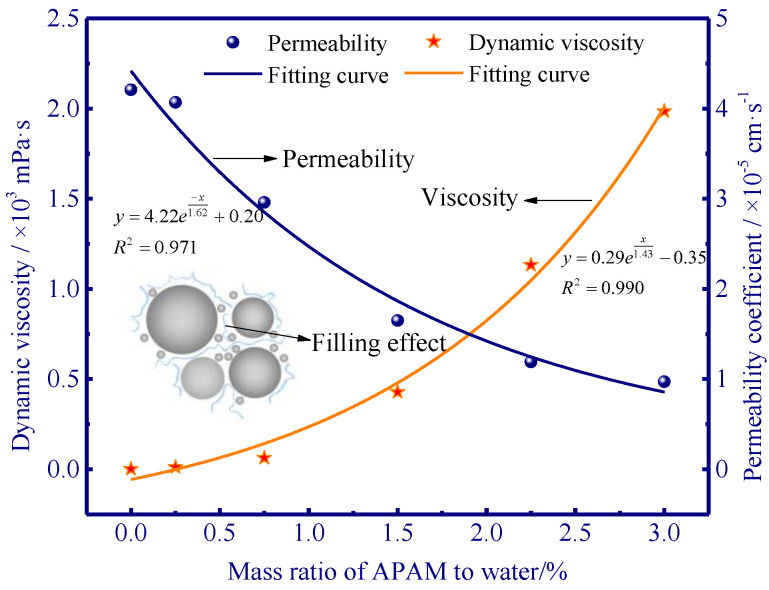
Viscosity and permeability with respect to different contents of APAM.

Exponential functions were used for curve fitting of viscosity and permeability experiments, and the R-squared values for each curve were 0.990 and 0.971, respectively (Figure 8). It is noted that the viscosity of APAM solution may have a major impact on water flow in seepage channels [34], and thus it can inhibit the migration of external water to the bottom of the ice lens. The APAM solution was influenced by the APAM content, and it significantly thickened for the APAM concentrations higher than 1.5% (Figure 8). This can increase the resistance of water migration and cause a decrease in the water migration rate and frost heave rate. However, the variation of permeability demonstrated a decreasing trend. The permeability decreased as the APAM concentration increased, and the decreasing trend slowed down for the APAM concentrations exceeding 1.5% (Figure 8). This is probably due to the residue of APAM in the pores and the adsorption of APAM onto soil particles, which narrow the seepage channels and decrease the soil permeability.

### 3.2. SEM Results

**Figure 9:**
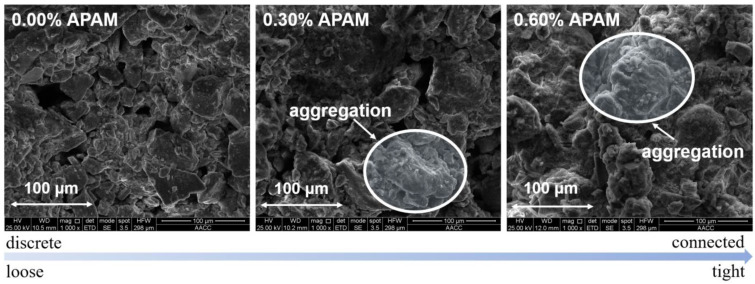
SEM images of soil particles with different contents of APAM.

SEM images of the APAM-modified soil (0.0%, 0.30%, and 0.60% APAM) after freezing were collected for the mineral morphology analysis. In the control experiment (0.0% APAM), the soil particles were randomly and discretely distributed, and loose assemblies of particles were observed (Figure 9). With the increase in APAM content, soil particles were interconnected and cemented with each other, and the aggregation of soil particles was therefore observed. This implies that the addition of APAM can potentially promote the cementation of different particles [35], thus enhancing the cohesion of soil particles and inhibiting ice lens formation. Moreover, the viscous fluid film could be adsorbed onto the soil particle surfaces. The adsorbed liquid film can narrow the seepage channels for water migration, but it may also increase migration resistance due to the increased dynamic viscosity (Figure 8). This poses an adverse effect on ice lens growth and frost heave.

### 3.3. FTIR Results

**Figure 10:**
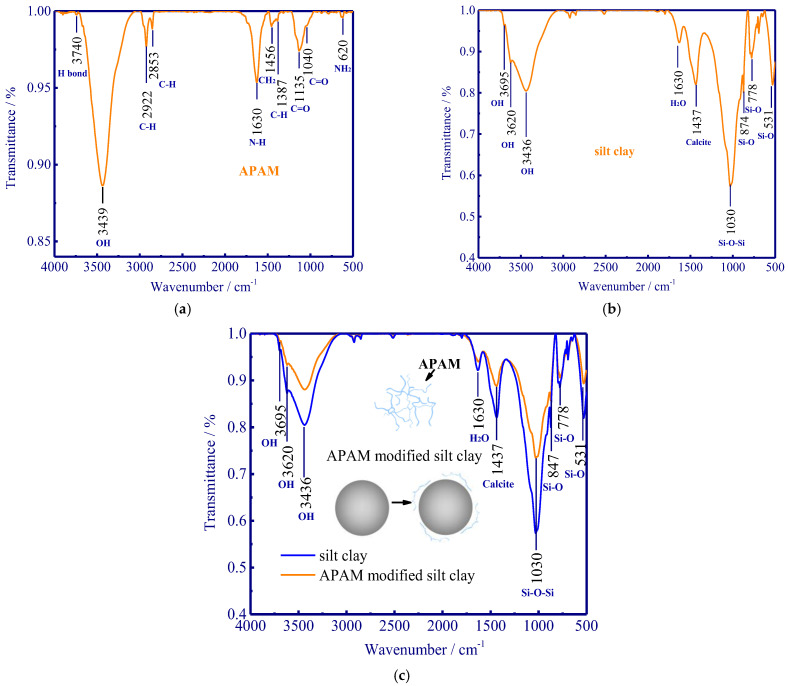
FTIR results of APAM, silty clay, and APAM-modified silty clay. (**a**) FTIR of APAM; (**b**) FTIR of silty clay; (**c**) FTIR of APAM-modified silty clay.

FTIR is employed to identify functional groups in specific substances, and this method is based on vibrational bonds between atoms and can enable a fundamental understanding of the adsorption mechanism of APAM onto silty clay (in the range between 4000 cm^−1^ to 400 cm^−1^). The N-H and C=O can induce the formation of hydrogen bonds in APAM, and the corresponding peak of a hydrogen bond occurs at 3740 cm^−1^ (Figure 10a). The peak at 3439 cm^−1^ is attributed to OH stretching vibration. The C-H asymmetric and symmetric stretching vibrations are identified by the peaks at 2853 cm^−1^ and 2922 cm^−1^, respectively. In addition, the N-H, CH_2_, and C-H bending vibrations are identified by the peaks at 1630 cm^−1^, 1456 cm^−1^, and 1387 cm^−1^, and the peak at 1040 cm^−1^ represents C=O stretching vibration [36].

The peaks at 3695 cm^−1^ and 3620 cm^−1^ denote the stretching vibration of the hydroxyl group (Figure 10b). Moreover, the antisymmetric stretching vibration of Si-O-Si can be identified by the peak at 1030 cm^−1^. The peaks at 874 cm^−1^ and 778 cm^−1^ represent the stretching vibration of Si-O, whereas the peak at 531 cm^−1^ denotes the bending vibration peak of Si-O. Calcite is also found in silty clay with a characteristic peak of 1437 cm^−1^ (Figure 10b) [37].

In comparison to the APAM-free soil, the hydroxyl peak intensity of APAM-modified soil weakens at the peak of 3695 cm^−1^ and 3620 cm^−1^ since the hydroxyl group in montmorillonite layer can form hydrogen bonds with the hydrogen atom in the APAM (Figure 10c). Furthermore, the stretching vibration intensity of Si-O-Si (1030 cm^−1^) and the bending vibration intensity of Si-O (874 cm^−1^ and 778 cm^−1^) decrease compared to that of APAM-free soil. This is due to the formed hydrogen bonds between the oxygen atom in Si-O (in soil) and the hydrogen atom in N-H (in APAM), thus weakening the vibration of Si-O [37]. Given there are no newly formed peaks in APAM-modified soil, we can extrapolate that the hydrogen bonding dominated physical adsorption mainly controls the interaction between APAM and silty clay.

## 4. Discussion

**Figure 11:**
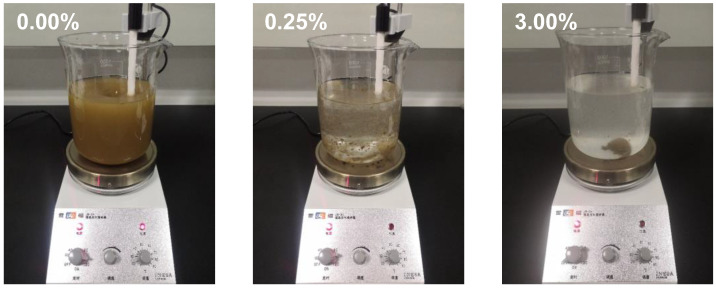
Photographs of dispersed soil particles in water without APAM compared to those in water with APAM.

The addition of APAM results in a decrease in the frost heave of silty clay (Figure 4). We interpret that the adsorption of APAM onto the soil particles’ surface slows down the water migration and the ice lens growth near the frozen fringe. Moreover, the dispersed soil particles in water without APAM compared to those in water with APAM demonstrated the adsorption between soil particles and APAM polymer (Figure 11). In the APAM polymer-free solution, the soil particles uniformly dispersed in the beaker (on a hot plate stirrer at room temperature), and a cloudy solution was then observed. However, in the APAM polymer solution (concentration of 0.25% and 3.00%), soil particles tended to aggregate, and the solution remained clear (Figure 11). The aggregation of soil particles enhanced as the APAM concentration increased, and a clearer solution is then observed. This visualises the aggregation of soil particles, and it corresponds to our SEM results in Figure 9.

**Figure 12:**
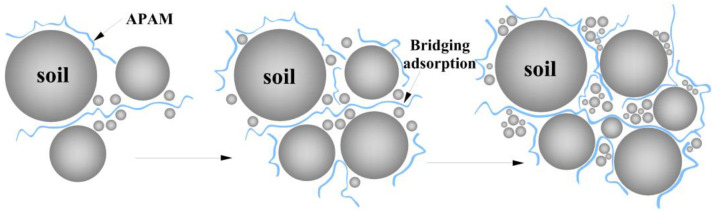
Bridging adsorption between APAM polymer and soil particles.

We highly speculate that a bridging adsorption could occur between APAM polymer segments and a particle surface. Bridging adsorption which can accumulate two or more soil particles in the presence of APAM polymer should be responsible for the aggregation of soil particles in the solution (Figure 12) [38]. This enables the possibility of attachment of APAM polymer segments to other particles and thus bridges solid particles together [39]. Hence, the bridging adsorption (Figure 12) potentially drives the aggregation of solid particles, and this explains what can be seen in Figure 11.

**Figure 13:**
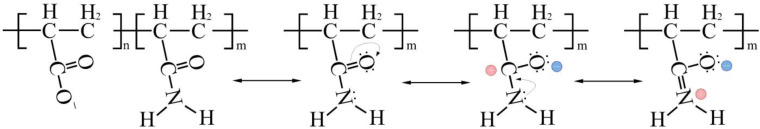
Schematic of electron delocalisation in an amide functional group (revised after [40]).

The adsorption of APAM polymer onto soil particles is highly likely via hydrogen bonding, and similar physical processes have been reported during the adsorption of APAM polymer on soil particles [40]. The hydrogen atoms (on the amide groups, Figure 13) and oxygen atoms on the soil particle surface can generate hydrogen bonding. It is noted that the carbonyl (C=O) group has a strong electron-withdrawing character, and this allows the migration of non-bonded electrons from nitrogen (N) to the adjacent carbonyl group (C=O) [39]. This can therefore increase the electron density around oxygen and pull the electron away from the hydrogen at the end of amide groups. Hence, a stronger positive dipole moment can be expected to occur in hydrogen atoms (in amide groups), suggesting intramolecular charge delocalisation may enhance the adsorption of PAM onto soil particle surface (compared to water). Furthermore, as a long-chain polymer, if one amide group forms a hydrogen bond with the soil particles, then all the other amide bonds in the chain will already be close to the soil particles, and it will be easier for them to be pulled closer to continue forming further hydrogen bonds and promoting particle aggregation through van der Waals attraction. However, the surface of the soil material is usually negatively charged in a normal pH range of 5 to 9, which prevents the adsorption of anionic polyacrylamide onto the soil surface because of the electrostatic repulsion [40]. Although the electrostatic repulsion may have a negative impact on the adsorption of APAM, our results confirm the aggregation of soil particles (Figure 11) and indicate that hydrogen bonding dominantly controls the adsorption of APAM onto soil particle surfaces (Figure 13). Furthermore, we speculate that APAM polymer is preferentially attached to the adsorption sites compared to water molecules (on soil particle surfaces), and a larger extent could be expected as the APAM concentration increases. The adsorption of APAM polymer will be enhanced by cooperative networks of hydrogen bonds since they all work together to make a stronger connection, but water molecules are more likely to be bonded individually and are more easily broken. Therefore, APAM can provide soil particles with a kind of surface coating as a result of APAM preferentially attaching to adsorption sites on mineral surfaces compare to water molecules.

The addition of APAM polymer decreases the frost heave of silty clay (Figure 4), and this is mainly due to the aggregation of soil particles (Figure 9 and Figure 11) and the enhanced soil cohesion via hydrogen bonding. Our results indicate that APAM polymer can increase soil cohesion (Figure 7), thus implying an increased difficulty in the formation of the ice lens since the ice lens only emerges if the disjoining pressure (occurring near the solid particle) exceeds the resistance of soil cohesion [6]. Moreover, APAM polymer thickens the fluid, thus potentially increasing the migration resistance of water. This can also slow down the water migration rate, thus delaying water accumulation at the bottom of the ice lens and inhibiting the growth of the ice lens. Hence, less water was observed to be redistributed with increasing APAM content (Figure 6). Less water redistribution is often reported after using physical and chemical treatment methods in frost heave mitigating fields [41,42]. Given the adsorption of APAM may narrow the seepage channels, we speculate that the pore blocking is the main reason for the decreased permeability (Figure 8). Reduction in soil permeability is also a critical pathway that can decrease frost heave, and similar findings can be found in previous research [43,44]. These results enable a fundamental understanding of the decreased frost heave with the addition of APAM polymer.

**Figure 14:**
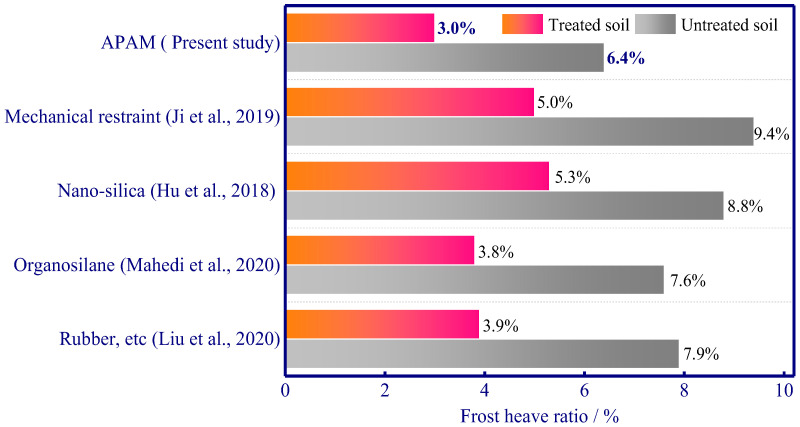
Frost heave ratio for this study and comparison with data from other studies [12,27,31,47].

Moreover, different researchers consider using other methods for frost heave mitigation [12,45,46,47]. Given different experiments may have different temperature conditions, different soil types, and different experimental procedures, we can only roughly evaluate the effect of different additives on the frost heave ratio (frost heave divided by freezing depth) of treated and untreated soil (Figure 14). Previous works also demonstrate mechanical restraint, nano-silica, organosilane, and rubber are effective in frost heave mitigation, and the estimated minimum frost heave ratio is 5.0%, 5.3%, 3.8%, and 3.9%, respectively. Our study shows that 0.60 wt% APAM can decrease the frost heave ratio from 6.4% to 3.0%. Hence, it can be found that the use of APAM polymers can considerably decrease frost heave. It is noted that mechanical restraint can inhibit ice lens formation, thus subsequently mitigating frost heave to 5.0% [45]. Moreover, 5.0% nano-silica (average particle size 15 nm) was reported to be effective in mitigating frost heave since it results in pore blocking and blocking the seepage channels (mercury intrusion porosimetry tests showed that porosity gradually decreased as nano-silica content increased), and this nano-additive can decrease frost heave ratio from 8.8% to 5.3% [46]. Organosilane (a silicon-based coupling agent, silica-based 10:1 organosilane solution was used to treat the soil at the addition content of 50% by weight) enables the formation of a hydrophobic soil, which can enhance water repellence capacity and reduce the freezing impacts on subgrade soils and decrease the frost heave ratio from 7.6% to 3.8% [12]. A rubber–asphalt–fibre mixture (capillary-break effect and smaller elastic modulus of the mixture were proven to contribute a smaller frost heave) was used to treat frost susceptible soil, and the optimum composition enabled a significant decrease in frost heave (a significant frost heave ratio reduction can be observed, frost heave ratio decreased from 7.9% to 3.9% by using a rubber–asphalt–fibre mixture) [47]. In addition, a lesser extent of the frost heave ratio (3.0%) in our study can be achieved using APAM polymers, and this validates its reliability in mitigating frost heave in freezing soil (Figure 14).

## 5. Conclusions

Freezing experiments were carried out to evaluate the frost heave mitigation and the underlying physicochemical mechanism with different anionic polyacrylamide content. We have documented that the kinetically decreased frost heave ratio is closely related to the chemically modified mineral-fluid interfaces. A better knowledge of the effect of APAM polymer can enable suitable and manipulated technologies in frost heave mitigation. The fundamental results of frost heave using APAM polymer can be used to define effective solutions for frost heave treatment when constructing road/railway subgrade in cold regions. Our main results are:

(1) APAM polymers significantly slow down frost heave velocity, and less water was observed redistributed in the soil column with increasing APAM content (the maximum water content near the frozen fringe decreases from 53.4% to 31.4% as APAM content increases from 0.0% to 0.60%). Frost heave is mitigated with the addition of APAM polymer, and 0.60% APAM can slow down the frost heave by a factor of 2.1 (8.56 mm divided by 4.14 mm).
(2) The adsorption of APAM onto soil particles originated from hydrogen bonding-induced physical adsorption (FTIR results demonstrate that no newly formed peaks are observed), and 0.60% APAM contributes a 20 kPa increase in cohesion force. The bridging adsorption can occur among the soil particles via hydrogen bonding, thus facilitating the aggregation of soil particles (enhancing soil cohesion) and causing difficulty in the formation of the ice lens.
(3) The addition of APAM can thicken the fluid and narrow seepage channels, thus enhancing the fluid viscosity and decreasing soil permeability. The viscosity increases from 0.0 to 2.0 × 10^3^ mPa·s as APAM content increases from 0.0% to 0.30%, and the corresponding soil permeability decreases from 4.2 to 1.0 × 10^−5^ $\mathrm{cm}\cdot s^{-1}$. These processes inevitably inhibit the feed of external water to the bottom of the ice lens and affect the growth of the ice lens (frost heave).

## Figures and Tables

**Table 1:** Mineralogical composition of the soil from Tibetan Plateau.

Quartz	Illite/Smectite	Albite	Muscovite	Chlorite
21%	52%	9%	10%	8%

**Table 2:** Physical properties of the soil.

Soil Type	Plastic Limit (%)	Liquid Limit (%)	Plastic Index	Water Content (%)	Dry Density (g/cm^3^)
Silty clay	17.2	25.2	8	25	1.6

## Data Availability

Not applicable.
